# On the Anticataractogenic Effects of L-Carnosine: Is It Best Described as an Antioxidant, Metal-Chelating Agent or Glycation Inhibitor?

**DOI:** 10.1155/2016/3240261

**Published:** 2016-10-16

**Authors:** Hamdy Abdelkader, Michael Longman, Raid G. Alany, Barbara Pierscionek

**Affiliations:** ^1^Faculty of Science, Engineering and Computing, Kingston University London, Penrhyn Road, Kingston upon Thames KT1 2EE, UK; ^2^Department of Pharmaceutics, Faculty of Pharmacy, Minia University, Minia, Egypt; ^3^School of Pharmacy, The University of Auckland, Auckland, New Zealand

## Abstract

*Purpose.* L-Carnosine is a naturally occurring dipeptide which recently gained popularity as an anticataractogenic agent due to its purported antioxidant activities. There is a paucity of research and conclusive evidence to support such claims. This work offers compelling data that help clarify the mechanism(s) behind the anticataract properties of L-carnosine.* Methods.* Direct in vitro antioxidant free radical scavenging properties were assayed using three different antioxidant (TEAC, CUPRAC, and DPPH) assays. Indirect in vitro and ex vivo antioxidant assays were studied by measuring glutathione bleaching capacity and total sulfhydryl (SH) capacity of bovine lens homogenates as well as hydrogen-peroxide-stress assay using human lens epithelial cells. Whole porcine lenses were incubated in high galactose media to study the anticataract effects of L-carnosine. MTT cytotoxicity assays were conducted on human lens epithelial cells.* Results.* The results showed that L-carnosine is a highly potent antiglycating agent but with weak metal chelating and antioxidant properties. There were no significant decreases in lens epithelial cell viability compared to negative controls. Whole porcine lenses incubated in high galactose media and treated with 20 mM L-carnosine showed a dramatic inhibition of advanced glycation end product formation as evidenced by NBT and boronate affinity chromatography assays.* Conclusion.* L-Carnosine offers prospects for investigating new methods of treatment for diabetic cataract and any diseases that are caused by glycation.

## 1. Introduction

Cataract is a primary cause of blindness worldwide and is one of the main causes of visual impairment in the elderly [1, 2]. The only available treatment is surgical extraction of the cataractous lens and replacement with an intraocular implant. This approach though tried and tested is not without limitations and complications [3, 4] such as endophthalmitis, retinal detachment, and recurrent posterior capsular opacity [5]. Cataract surgery remains difficult to access for some patients in developing countries and given the rise in population age, even in developed countries numbers of patients and consequently waiting lists for surgery are increasing. There is an underlying need to gain a far better understanding of the process of cataractogenesis.

Genetics, ageing, and certain chronic diseases such as diabetes have been recognised as risk factors for cataract [6]. Mechanisms of development of some rarer forms of cataract are better understood: Galactosaemia and Wilson's disease are two genetic diseases associated with lens opacification and these arise from genetic abnormalities of galactose metabolism and copper ion transport, respectively [6]. Processes underlying the most common forms of cataracts, senile cataract and diabetic cataract, require further research as the causes can be multifactorial.

The unique growth pattern of the human lens and accrual of new lens fibers onto existing cell layers coupled with no protein turnover and an age-related depletion of *α*-crystallin chaperone function render the human lens vulnerable to posttranslational modifications such as phosphorylation, glycation, deamidation, and aggregation which are caused by oxidative stress and decline of antioxidant enzymes [7–9]. These morphological and biochemical changes occur throughout life, progressing at a very slow rate. Cataract can also develop as a secondary effect of diabetes. The exact mechanism for diabetic cataractogenesis is still not understood but high levels of plasma glucose that allow glucose molecules to enter the lens independently are thought to be a major risk factor, leading to oxidative stress, formation of Schiff's base (a nonenzymatic reaction of glucose with residual primary amino groups of lens crystallins), and an accumulation of advanced glycation end products leading to yellow discoloration/browning of the lens [10]. It has been reported recently that there is a direct correlation between plasma glycated haemoglobin (HbA_1C_) levels and diabetic cataract. An estimate has been made that a 1% reduction of HbA_1C_ level is associated with at least 19% reduction of the risk of developing diabetic cataract [11]. This direct relationship can be attributed to increasing glucose entry into the lens causing glycation of lens proteins and antioxidant enzymes with a concomitant rise in copper levels [12]. Advance glycation end products have been reported to be able to bind/chelate copper [13, 14]. Recent reports highlight the importance of chelating agents in therapies that delay or prevent the formation of cataracts [15, 16]. Diabetics can develop lens brunescence and cataract at an earlier age than nondiabetics [17] and they are at greater risk of developing other postsurgical complications such as retinopathy, macular oedema, and endophthalmitis [11]. With increasing numbers of diabetic patients worldwide and lack of accessibility to surgical treatment of cataract in many countries, there is a pressing need to find effective nonsurgical treatments that can delay or reverse diabetic cataract.

L-Carnosine is marketed as a prodrug in the form of N-acetylcarnosine (NAC) eye drops (1%). N-Acetyl carnosine was found to have a dose-dependent hydrolysis releasing carnosine into the anterior chamber 15–30 minutes after instillation onto ocular surface of rabbit lenses [18]. These eye drops have been considered to be promising with respect to treatment/prevention of senile cataract in human eyes and showed good ocular tolerability for up to 6 [18] and 9 [19] months. Topical application of NAC (2% w/w) showed improvements in lens opacities of canine immature cataracts or nuclear sclerosis, whereas a marginal reduction was recorded for mature cataract or cataracts associated with intraocular inflammation [20]. However, the exact mechanism is still unknown and the Royal College of Ophthalmologists issued a public statement in August 2008 to indicate that there is insufficient evidence to support the potential of this drug for cataract treatment [21].

L-Carnosine is a *β*-alanyl histidine dipeptide that is endogenously present in appreciable amounts in skeletal muscles, in the lens, and in relatively lower concentrations in other human tissues [22]. Since its discovery in 1900, numerous therapeutic benefits have been attributed to the exogenous administration of L-carnosine; among these are antiageing, anticancer, and anticataractogenic properties [23–25]. Its exact physiological function is still unknown; nevertheless, enhancing buffering capacity of the cell, the recovery from muscle fatigue, and membrane stabilizing effect are the main identifiable metabolic roles ascribed to this histidine dipeptide [22]. Antiglycating, antioxidant, and antiproliferative properties have been also reported for L-carnosine. Whilst antiglycating properties have been reported in recent publications [24, 26, 27], the antioxidant properties are inconclusive and lack sufficient support. Indirect antioxidant properties were also claimed for L-carnosine in an attempt to explain how L-carnosine could work clinically. These indirect antioxidant effects have been reported due to possible in vitro chelating properties of copper and there might be possibilities of inhibiting the initiation of intracellular Fenton's reaction. L-Carnosine did not show any oxidation by a common oxidizing agent such as hydrogen peroxide [28]. This work is aimed at investigating possible antioxidant, antiglycation, and copper chelating effects of L-carnosine; further, this work is interested in studying the cytotoxicity of L-carnosine.

## 2. Materials and Methods

### 2.1. Materials

L-Carnosine, aminoguanidine HCl, sodium azide, nitro blue tetrazolium (NBT), 4-(2-hydroxyethyl)-1-piperazineethanesulfonic acid (HEPES), copper (II) chloride, sorbitol, ammonium acetate, *α*-crystallin from bovine eye lens, and 3-(4,5-dimethylthiazol-2-yl)-2,5-diphenyltetrazolium bromide (MTT) were purchased from Sigma-Aldrich, UK. Ascorbic acid, glutathione, and 2,2′-azino-bis-(3-ethylbenzothiazoline-6-sulfonic acid) (ABTS) were purchased from TCI, Toshima, Tokyo, Japan. 6-Hydroxy-2,5,7,8-tetramethylchroman-2-carboxylic acid (Trolox) was purchased from Acros Organics, New Jersey, USA. 2,9-Dimethyl-1,10-phenanthroline (neocuproine) and 5,5′-dithiobis (2-nitrobenzoic acid) (Ellman's reagent) were purchased from Alfa Aesar, Heysham, UK. All other chemicals and reagents were of analytical grade and used as received.

### 2.2. Antiglycating Assay

A modification of the method reported by Matsuda et al. [29] which is based on measuring the characteristic fluorescence of the advanced glycation end products (AGEs) was used. In brief, 100 mg/mL D-glucose and 10 mg/mL of porcine lens proteins (PLP) were separately prepared in phosphate buffer (67 mM) pH 7.2 containing 0.02% sodium azide as antimicrobial preservative. Equal volumes of the glucose and PLP solutions were mixed without and with different concentrations (10 mM and 20 mM) of L-carnosine and aminoguanidine (a standard antiglycating agent). The solutions without L-carnosine and aminoguanidine acted as positive controls because they do not contain antiglycating agents. Two negative controls for L-carnosine and aminoguanidine were used: equal volumes of glucose (100 mg/mL) with different concentrations of L-carnosine (to eliminate the sacrificing glycation due to reaction of L-carnosine with glucose) and equal volumes of PLP (10 mg/mL) and aminoguanidine without glucose, respectively. All samples were incubated for 14 days at 40°C. A volume of 20 *μ*L was withdrawn from each sample and transferred into a 96-well plate. An aliquot of 180 *μ*L of the phosphate buffer was added to each well and the intensity of fluorescence was measured at excitation and emission wavelengths of 370 nm and 440 nm, respectively, using a TECAN Infinite M200 Pro plate reader (Männedorf, Switzerland). Percentage inhibition of AGEs formation was estimated using

(1) $$\begin{array}{c} AGEs=\frac{I_{p}-\left(I_{s}-I_{c}\right)}{I_{p}}*100, \end{array}$$

where *I*_p_, *I*_s_, and *I*_c_ are fluorescence intensities of the positive control, sample, and corresponding negative control (S1).

### 2.3. Antioxidant Assays

#### 2.3.1. Trolox Equivalent Antioxidant Capacity (TEAC) Assay

This assay is based on quenching of the blue-green colour of the stable free radical 2,2′-azino-bis(3-ethylbenzothiazoline-6-sulphonic acid (ABTS^∙^) by the tested antioxidant. ABTS^∙^ was generated by the reaction between 7 mM ABTS and 2.45 mM potassium persulphate in water. The solution was stored in the dark for 12–16 hours before use. The concentration of the generated ABTS^∙^ was diluted with phosphate buffered saline (PBS) pH 7.4 to give a final absorbance of 0.70 ± 0.02 at 734 nm. A concentration of 500 *μ*M Trolox in PBS was prepared and different concentrations of L-carnosine (up to 20 mM) were tested. Additional samples of ascorbic acid and glutathione were used as validation samples. These two antioxidants are chosen because they are endogenously available in aqueous humour and in human lens. Aliquots of 20 *μ*L each of PBS, L-carnosine, ascorbic acid, glutathione, and Trolox were added to 980 *μ*L of ABTS^∙^ in Eppendorf tubes and absorbance was measured at 734 nm. Antioxidant scavenging capacity and TAEC were estimated through (2) and (3), respectively:

(2) $$\begin{array}{c} \text{Scavenging }{Capacity}_{\text{sample}}={\text{OD}}_{\text{control}}-{\text{OD}}_{\text{sample}}, \end{array}$$

where OD_control_ and OD_sample_ are the optical density of ABTS^∙^ after addition of 20 *μ*L of PBS and sample, respectively,

(3) $$\begin{array}{c} TEAC=\frac{\text{Scavenging }{\text{capacity}}_{\text{sample}}}{\text{Scavenging }{\text{capcity}}_{\text{trolox}}}. \end{array}$$

#### 2.3.2. CUPRAC Antioxidant Assay

Aqueous solutions of copper (II) chloride (0.01 M), ammonium acetate (1 M), and alcoholic (ethanol 96%) solution of neocuproine (7.5 mM) were prepared and mixed in a glass vial in the following order: 1 mL of copper (II), 1 mL of neocuproine, 1 mL of ammonium acetate, and 1 mL of antioxidant solution [30]. The final mixture was allowed to stand for 30 min at room temperature and absorbance was measured at 450 nm. TEAC was estimated by dividing OD of the sample by OD of Trolox.

#### 2.3.3. Diphenylpicrylhydrazyl (DPPH) Assay

The DDPH assay was performed as previously reported [29]. In brief, equal volumes of DPPH (100 *μ*M) solution and antioxidant solutions were mixed and absorbance was measured at 517 nm. TEAC for L-carnosine, ascorbic acid, and glutathione was determined as mentioned above.

### 2.4. Indirect Antioxidant (Metal-Chelating Antioxidant) Assays

#### 2.4.1. Bleaching Capacity of Glutathione and Glutathione-Copper Complex with and without L-Carnosine

Antioxidant capacities of glutathione alone, glutathione : copper (II) (1 : 1 mol/mol), glutathione : copper (II) : L-carnosine (1 : 1 : 1 and 1 : 1 : 2 mol/mol/mol), and glutathione : copper (II) : EDTA (1 : 1 : 1 mol/mol/mol) were measured. The bleaching capacity (indication of antioxidant capacity) was measured as mentioned in Section 2.3.1 and determined using (1).

#### 2.4.2. Sulfhydryl Capacity of Glutathione and Glutathione-Copper Complex with and without L-Carnosine

The sulfhydryl capacity of glutathione alone and glutathione : copper (II) (1 : 1 mol/mol), glutathione : copper (II) : L-carnosine (1 : 1 : 1 and 1 : 1 : 2 mol/mol/mol), and glutathione : copper (II) : EDTA (1 : 1 : 1 mol/mol/mol) was estimated using Ellman's reagent [31]. In brief, an aliquot of 250 *μ*L of Ellman's reagent 0.4% in Tris-HCl buffer pH 8.4 was added to a premixed solution of 200 *μ*L of crystallin sample and 750 *μ*L of Tris-HCl buffer/pH 8.4. The final mixture was allowed to stand for 10 minutes at room temperature and the generated yellow colour is measured at 412 nm. A blank solution was made up of the sample volumes by replacing 200 *μ*L crystallins with 200 *μ*L Tris-HCl buffer.

### 2.5. Total Antioxidant Capacity of the Lens Homogenate Incubated with High Levels of Copper (II)

Fresh excised bovine eyes were collected from a local abattoir (ABP Guildford Slyfield, Surrey, UK) and lenses were extracted within 10 hours of death. Whole lenses were homogenised in a flacon tube with 12 mL of Tris-HCl buffer pH 8.4 containing 0.02% sodium azide. The homogenate was centrifuged at 10,000 rpm for 30 minutes at 4°C. The supernatant was collected to obtain water soluble (WS) protein fraction [32].

The soluble protein solutions were mixed with aqueous solution s of copper (II) with and without L-carnosine (20 mM) to form final concentrations of copper (II) 100 *μ*M, 10 *μ*M, and 1 *μ*M. Samples of lens crystallins without copper (II) were used as control. The total sulfhydryl capacity of lens crystallins was estimated using Ellman's reagent as mention in Section 2.4.2.

### 2.6. Protection of Human Lens Epithelial Cells from H_2_O_2_-Induced Damage by L-Carnosine

B-3 human lens cells (ATCC CRL-11421) were grown in accordance with supplier instructions. Briefly, cells were prepared and seeded out into 96-well plates (Nunclon Delta, Nunc, Netherland) at approximately 2 × 10^4^ cells/well in Eagle's Minimum Essential Medium (EMEM) containing 20% foetal bovine serum (LGC standards). The cells were allowed to establish for 48 hours prior to treatment. Media were subsequently removed and fresh media containing treatments were added; 8 wells were used per treatment condition and all experiments were performed in triplicate. Treatments were two different concentrations of L-carnosine (10 mM or 20 mM final concentration), in the presence or absence (negative control) of either 1 mM or 0.25 mM hydrogen peroxide (final concentration, positive controls) or untreated for two durations: 1 hour and 24 hours. After treatment duration, the media were removed and the cells were washed twice with 37°C sterile PBS. The cells were then incubated with 200 *μ*L per well of 0.5 mg/mL 3-(4,5-dimethylthiazol-2-yl)-2,5-diphenyltetrazolium bromide (MTT) solution in 37°C EMEM (LGC standards) with no further additions of serum for 3 hours at 37°C. After incubation, the MTT solution was carefully removed and the wells were washed twice with sterile PBS. Finally, 200 *μ*L of dimethylsulfoxide (DMSO) was added to each well to lyse the cells. The cells were then gently agitated to mix the samples and analyzed on a TECAN Infinite M200 Pro plate reader using a wavelength of 540 nm.

### 2.7. Incubation of the Lens in High Sugar Media with and without L-Carnosine

Fresh excised porcine eyes were collected from a local abattoir and the lens was removed within less than 24 hours postmortem. Lenses were rinsed in M199 (Sigma-Aldrich, UK) supplemented with 8% foetal bovine serum (Sigma-Aldrich, UK) and antibiotics (penicillin/streptomycin, 100 units/mL) (Sigma-Aldrich, UK) and transferred onto a 6-well plate (Nunclon*™* Delta Surface, Roskilde, Denmark) containing 10 mL of the medium and incubated for 12 hours at 35°C/5% CO_2_ [33]. Any lenses showing signs of opacity were discarded. The incubated lenses were divided into four groups: a negative control (lenses incubated in the culture medium without galactose), positive controls (lenses incubated in a medium containing 30 mM galactose), and two treated groups containing 10 mM and 20 mM of L-carnosine of media supplemented with 30 mM galactose. All incubated lenses were incubated at 35°C/5% CO_2_. Media were changed every 24 hours for 3 days. Optical integrity, lens transparency, and any opacification were evaluated visually by looking at a grid through the lens.

#### 2.7.1. Size Exclusion Chromatography and Estimating of *α*-Crystallins

After optical evaluation, lenses were homogenised in 8 mL Tris-HCl buffer pH 8.4 containing 0.02% sodium azide water soluble as described in Section 2.5. Water soluble (WS) fractions were prepared as mentioned above in Section 2.5 and separated into fractions based on size using HPLC (Shimadzu LC-2010AHT, Shimadzu Corporation, Kyoto, Japan) comprising a quaternary pump, an automatic sampler, and a UV (280 nm) detector with data acquisition (Lab solutions software version 5.42 SP5, Shimadzu Corporation, Japan) and a size exclusion column, Yarra-SEC 4000 (3 *μ*m; 300 mm × 7.8 mm, Phenomenex Corporation, UK), maintained at 40°C. The mobile phase comprised of 50 mM sodium phosphate pH 6.8/0.15 M sodium chloride. The isocratic flow rate was 1.0 mL/min. Purified *α*-crystallin was purchased from Sigma-Aldrich and used as a reference. The percentage (%) of soluble *α*-crystallin fraction was estimated as follows:

(4) $$\begin{array}{c} \alpha \text{-crystallin fraction }\left(\;\%\;\right)=\frac{{AUC}_{s}}{{AUC}_{c}}*100, \end{array}$$

where AUC_s_ and AUC_c_ are the area under the size exclusion (SE) chromatograms for *α*-crystallin fraction of the sample and negative control, respectively, and a representative chromatogram is shown as in Supplementary Figure 2 (in Supplementary Material available online at http://dx.doi.org/10.1155/2016/3240261).

#### 2.7.2. Nitro Blue Tetrazolium (NBT) Assay

This assay is based on the reducing properties of AGEs or better known as fructosamine to NBT to formazan (purple colour) under alkaline conditions [34, 35]. An aliquot of 100 *μ*L of WS fractions was added to 1 mL of NBT (0.3 M in carbonate buffer pH 10.35). The mixtures were incubated at 37°C for 10 min, allowed to cool down to room temperature, and measured spectrophotometrically at 530 nm. Percentage of glycated crystallins was estimated using

(5) $$\begin{array}{c} \%\;\text{Glycated crystallins}=\frac{{OD}_{s}-{OD}_{c}}{{OD}_{c}}*100, \end{array}$$

where OD_s_ was the optical density of the positive control or treated samples and OD_c_ is the optical density of the negative control.

#### 2.7.3. Boronate Affinity Chromatography for Separation of Glycated Proteins

An AKTA protein purification system (AKTA Purifier, GE Healthcare Bioscience AB, Uppsala, Sweden) comprising a binary pump and a UV detector with data acquisition (Unicorn software version 5.11, GE Healthcare Bioscience AB, Uppsala, Sweden) were used to separate glycated crystallins from nonglycated crystallins on a TSKgel Boronate 5PW (10 *μ*m; 75 mm × 7.5 mm, TOSOH Bioscience Corporation, Shiba, Japan) maintained at ambient condition. The binding buffer was comprised of 20 mM HEPES (pH 8.5) and 20 mM MgCl_2_. The elution buffer was comprised of 20 mM HEPES (pH 8.5) and 100 mM sorbitol. The isocratic flow rate used was 1.0 mL/min and the injection volume was fixed at 200 *μ*L. Detection was by UV absorbance at a wavelength of 280 nm.

#### 2.7.4. Cytotoxicity Evaluation (MTT Assay) of L-Carnosine Using Human Lens Epithelial Cells

B-3 human lens cells (ATCC CRL-11421) from ATCC were prepared and seeded out at approximately 2 × 10^4^ cells/well into 96-well plates (Nunc, Netherland) in Eagle's Minimum Essential Medium (EMEM) containing 20% foetal bovine serum (LGC standards). The cells were allowed to establish for 48 hours prior to treatment in the 96-well culture plate. Media were subsequently removed and fresh media containing treatments (8 wells used per condition) were added. The treatments were three different concentrations of L-carnosine 1%, 0.5, and 0.1% w/v. The untreated media were used as a negative control and hydrogen peroxide (300 mg/mL) and benzalkonium chloride (BKC) at a concentration of 0.01% w/v were used as positive controls. After 3 hours of treatment, the media were removed and the cells were washed twice with 37°C sterile PBS. The cells were then incubated with 200 *μ*L per well of 0.5 mg/mL 3-(4,5-dimethylthiazol-2-yl)-2,5-diphenyltetrazolium bromide (MTT) solution in 37°C EMEM (LGC standards) with no additions for 3 hours at 37°C. After incubation, the MTT solution was carefully removed and the measurements were performed as described in Section 2.6.

#### 2.7.5. Statistical Analysis

Statistical analysis was performed with GraphPad Prism 6 (2014) software, using analysis of variance (ANOVA) with a Dunnett post hoc test for confidence intervals of 95% with statistical significance set at *P* < 0.05.

## 3. Results and Discussion

### 3.1. Antiglycating Assay

**Figure 1:**
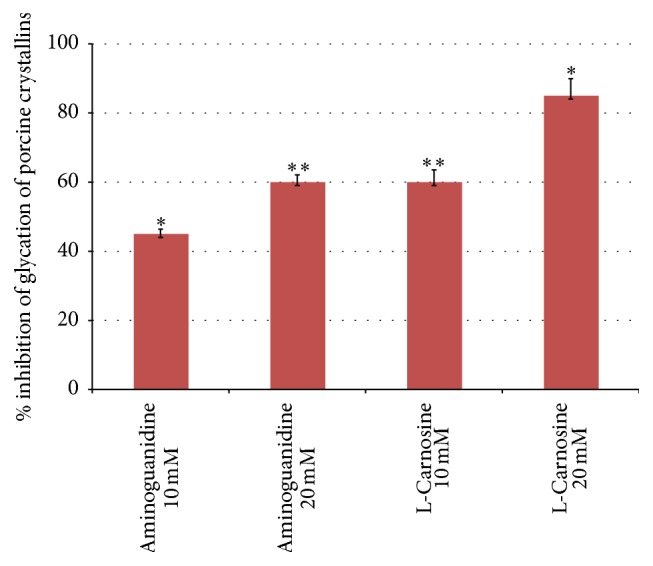
Comparative percentage inhibition of advanced glycation end products formation recorded for porcine lens crystallins with two equivalent concentrations (10 mM and 20 mM) of aminoguanidine and two different concentrations of L-carnosine. Results are presented as mean values ± SD [^*∗*^statistically significant; ^*∗∗*^not significant].

Figure 1 shows percentage inhibition of formation of advanced glycation end products (AGEs) with two different concentrations (10 mM and 20 mM) of aminoguanidine (a standard antiglycating agent) and two equivalent concentrations (10 mM and 20 mM) of L-carnosine. The results demonstrated that L-carnosine significantly inhibited glycation compared with aminoguanidine (*P* < 0.01) at equivalent concentration. For example, 80% inhibition was estimated for L-carnosine 20 mM, whereas 60% inhibition was recorded for an equivalent concentration of aminoguanidine.

L-Carnosine has a terminal amino group of the beta-alanine moiety with imidazole ring structure that can provide a more favourable position than *ε*-amino residues of crystallins for scavenging carbonyls and can be more readily glycated by aldose sugars [24, 26, 27]. This also was evident from fluorescence intensity measurements where the L-carnosine-glucose mixture showed significantly greater intensity compared with the lens crystallins-glucose (negative control) mixture (S1). These findings support the idea that L-carnosine behaves as an antiglycating agent. This effect was concentration dependent, as approximately 35% of antiglycation capacity increased by doubling the concentration of L-carnosine.

### 3.2. Antioxidant Assays

**Figure 2:**
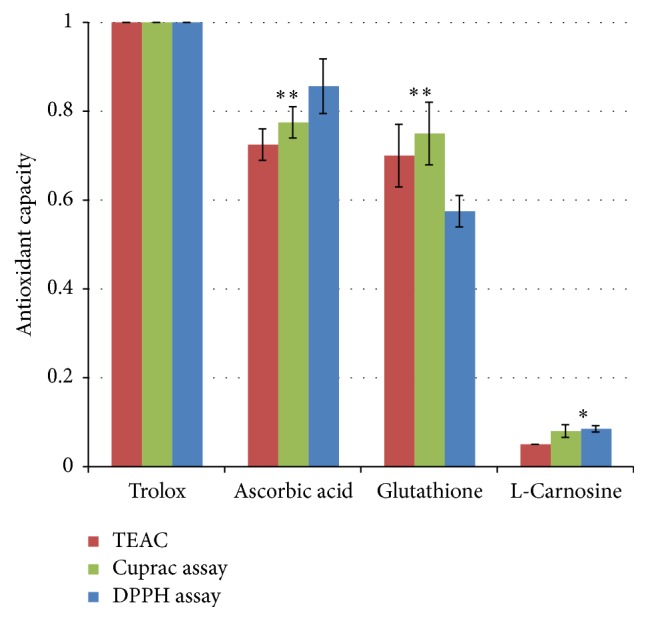
Trolox equivalent antioxidant capacity of L-carnosine compared with other endogenous antioxidants using three different antioxidant assays. Results are presented as mean values ± SD, *n* = 3 [^*∗*^statistically significant; ^*∗∗*^not significant].

The antioxidant capacity of L-carnosine was evaluated using different commonly used assays for assessing antioxidant capacity of many drug molecules, flavonoids, and foods [25, 36].  Figure 2 shows Trolox equivalent antioxidant capacity for ascorbic acid, glutathione, and L-carnosine using the abovementioned assays.

The results showed that L-carnosine exhibited very weak direct antioxidant capacity, compared with standard antioxidants: glutathione and ascorbic acid. Antioxidant capacity recorded for L-carnosine by measuring the bleaching of DPPH (1 mM) was only 2.5% compared with the bleaching capacity of 36% and 39% for the antioxidant bisdiaminotriazole compounds, namely, compounds 1 and 1, respectively [25].

### 3.3. Indirect Antioxidant (Metal-Chelating Antioxidant) Assays

**Figure 3:**
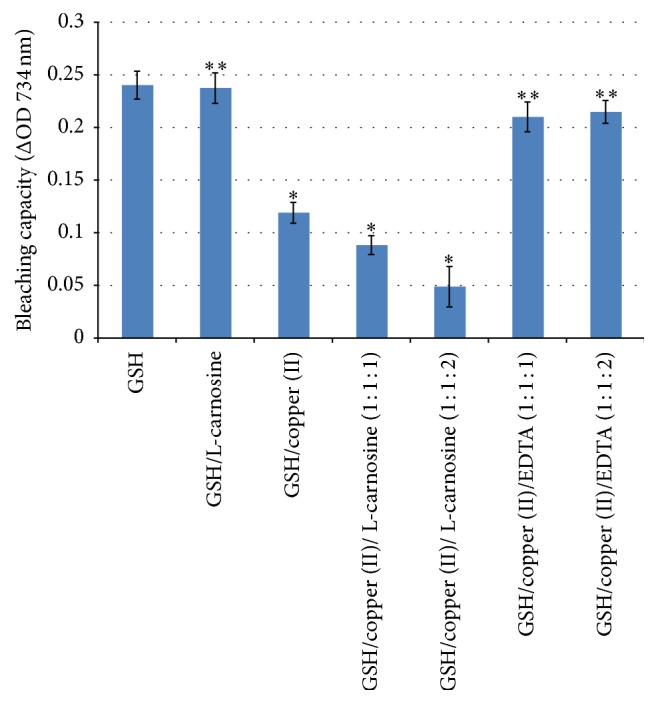
Bleaching (of the coloured free radicals ABTS^∙^) capacity of GSH alone and GSH with equimolar ratios of copper, L-carnosine, and EDTA. Results are presented as mean values ± SD, *n* = 3 [^*∗*^statistically significant; ^*∗∗*^not significant].

**Figure 4:**
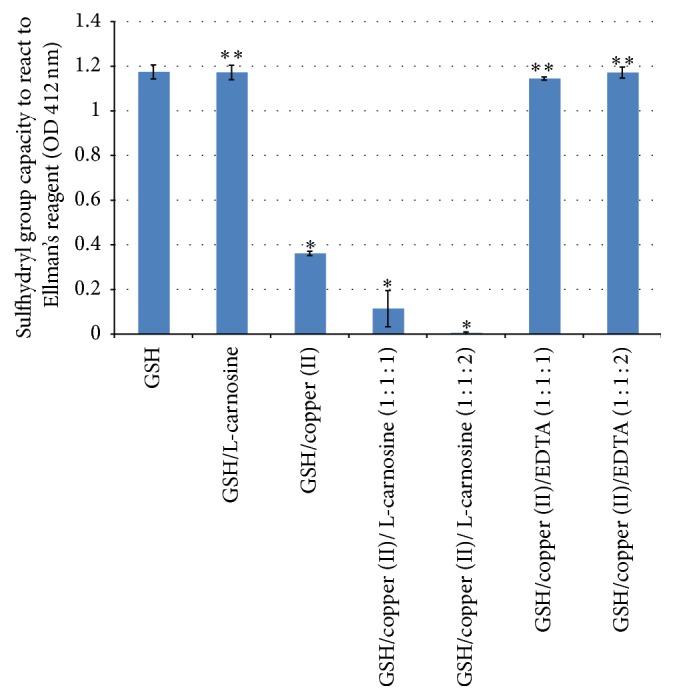
Sulfhydryl capacity of glutathione alone, glutathione with L-carnosine, glutathione with copper, and glutathione and copper with different molar ratios of L-carnosine and EDTA. Results are presented as mean values ± SD,  *n* = 3 [^*∗*^statistically significant; ^*∗∗*^not significant].

Other studies have found indirect antioxidant capacity for L-carnosine arising from the chelation of copper, inhibiting Fenton's reaction and free radical formations [22]. Reduced glutathione (GSH) is an endogenous antioxidant and it is available in appreciable concentrations in the human lens [36]. Decline of glutathione levels in the lens has been found to be associated with oxidation and disulfide cross-linking of lens crystallins and cataract formation [6]. This study explored whether there is any possible indirect antioxidant activity for L-carnosine by potentiating glutathione system. This hypothesis was studied by two different methods: firstly; measuring the fading (bleaching) of the blue-green colour of ABTS^∙^ due to reduction to ABTS by GSH and secondly assessing the sulfhydryl capacity of GSH under different conditions as shown in Figures 3 and 4.

Figure 3 shows the bleaching capacity of GSH under different conditions (GSH alone, GSH with L-carnosine, GSH with copper (II), GSH with copper (II), and L-carnosine at two different molar ratios and GSH, with copper (II) and EDTA). GSH shows the highest bleaching capacity (decolourising the free radicals ABTS^∙^) given that it is a potent antioxidant. Addition of L-carnosine did not show any significant potentiation of the bleaching capacity of GSH, indicating the lack of antioxidant capacity of L-carnosine which supports the results found with antioxidant assays. Addition of copper significantly reduced the antioxidant capacity of GSH (*P* < 0.05); this is likely to be caused by the chelating properties of GSH rather than the redox reaction [37]. Glutathione has been reported to have molecular chaperone properties for copper in the cytoplasm of the lens epithelial cells [37]. Consistent with this report, addition of a stronger chelating agent (EDTA) regenerates the natural bleaching capacity of glutathione (Figure 3).

Figure 4 shows sulfhydryl capacity of glutathione and glutathione treated with the six different conditions as described above. Significant depletion of sulfhydryl groups of GSH was recorded with the GSH samples containing copper alone and copper and L-carnosine, whereas sulfhydryl capacity was restored to normal levels after the addition of EDTA but not with L-carnosine. These results are similar to those reported with bleaching capacity (Figure 3) and this is also in support to the abovementioned results. These findings support the idea that L-carnosine does not show indirect antioxidant capacity.

### 3.4. Protection of Human Lens Epithelial Cells from H_2_O_2_-Induced Damage by L-Carnosine

**Figure 5:**
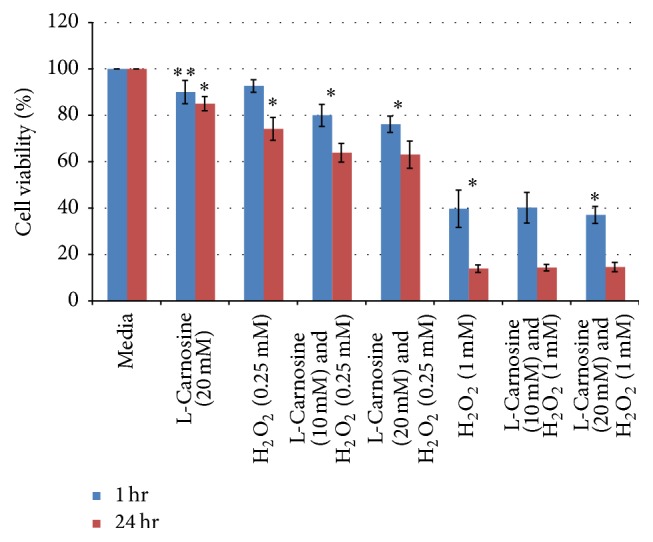
Human lens epithelial cell viability after exposure to two concentrations of H_2_O_2_ with and without two different concentrations of L-carnosine. Results are presented as mean values ± SD, *n* = 3 [^*∗*^statistically significant; ^*∗∗*^not significant].

These two concentrations (10 mM and 20 mM) of L-carnosine used to investigate whether this substance could protect cultured human lens epithelial cells from H_2_O_2_ were selected based on preliminary screening of different concentrations. The cells subjected to these two concentrations showed inhibited proliferation and significant oxidative damage compared with negative control cells. The possible protective mechanism of L-carnosine (10 mM and 20 mM) was studied over two different time points of 1 and 24 hours corresponding to any possible protection from either direct free radical scavenging or gene induction effect due to possible H_2_O_2_-induced damage to DNA, respectively (Figure 5).

The results showed that L-carnosine had no protective effect on cells from the oxidant activity of hydrogen peroxide. These findings are in line with the in vitro antioxidant assays as well as other results that have been recently reported elsewhere [25].

### 3.5. Total Antioxidant Capacity of the Lens Homogenate Incubated with High Levels of Copper

Total sulfhydryl capacity of lens has been an indirect indicator of oxidative stress and damage to lens crystallins [32]. Copper can catalyze free radical formation and reactive oxygen species (ROS) [22, 38]. In this study, copper was added to the lens homogenate to induce oxidative stress via Fenton's reaction.

**Figure 6:**
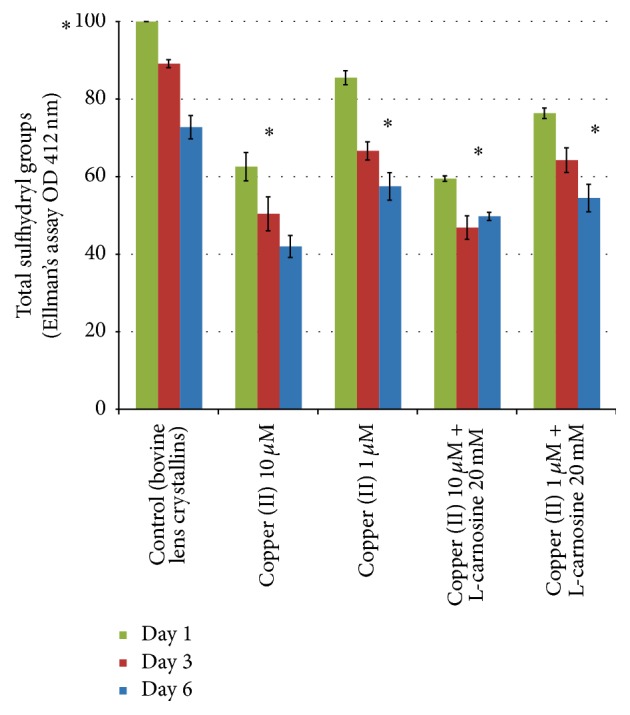
Total sulfhydryl capacity of lens homogenate incubated with different concentrations of copper with and without L-carnosine. Results are presented as mean values ± SD, *n* = 3 [^*∗*^statistically significant].

Figure 6 shows total sulfhydryl capacity of lens homogenate incubated with copper and L-carnosine for 6 days at 35°C. The presence of copper showed a concentration dependent depletion of sulfhydryl capacity; the addition of L-carnosine did not show any significant protection.

### 3.6. Lens Incubation in High Sugar Media

Induction of experimental cataract is an important approach used to gain a better understanding of cataract formation and to help develop potential therapeutic treatments that could delay or reverse cataractogenesis. To date, there is no in vivo or in vitro experimental model that can simulate human senile cataract in terms of the time required and physiological concentrations of cataract inducers because this form of cataract develops over many decades period with more than a single causal factor. Cataracts that occur as secondary manifestations of diabetes, however, can develop at faster rates. Numerous in vivo and in vitro models have been studied [39, 40]. Galactose is approximately 2.5 times more favourable as a substrate for aldose reductase than glucose and can cause substantially higher levels of osmotic stress and generate a larger number of free radicals, compared with glucose [41]. Galactose is also a significantly more active glycating agent than glucose. Galactose is available in 3.5 times higher concentration of the acyclic open (reactive) form, that is, with glucose at pH 7.5 [42]. Galactose was used in this study to induce cataract formation in vitro in excised bovine lenses.

On the third day of incubation, harvested lenses from negative control, positive control, and treated groups were placed on a grid to investigate optical quality. Lenses incubated in a high galactose medium showed marked yellowing/browning of lens cortex, compared with control lenses incubated in a medium free of galactose. Lens brunescence is a consequence of glycation and likely to mimic the processes that underlie diabetic cataract [13]. The lens brunescence was partially inhibited by supplementing the media with 10 mM L-carnosine; lenses incubated with 20 mM L-carnosine demonstrated a level of transmittance that appeared to be similar to that of negative controls. This concentration (20 mM) has been reported to be the minimum effective concertation of L-carnosine in the aqueous humour to produce anticataractogenic effects [22].

**Figure 7:**
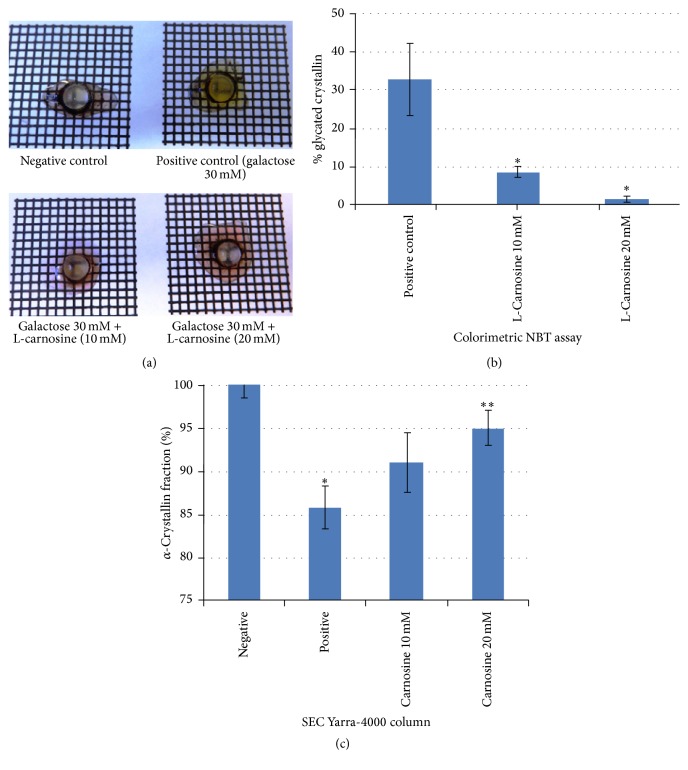
Porcine lenses incubated in negative control medium and in high galactose medium, high galactose with 10 mM L-carnosine, and high galactose with 20 mM L-carnosine (a), percentage of *α*-crystallin remaining after lens incubation measured for negative control, positive and treated lenses with L-carnosine (10 mM and 20 mM) (b), and percentage of glycated proteins for positive control and treated lenses with L-carnosine (10 and 20 mM) (c). Results are presented as mean values ± SD, *n* = 3 [^*∗*^statistically significant; ^*∗∗*^not significant].

**Figure 8:**
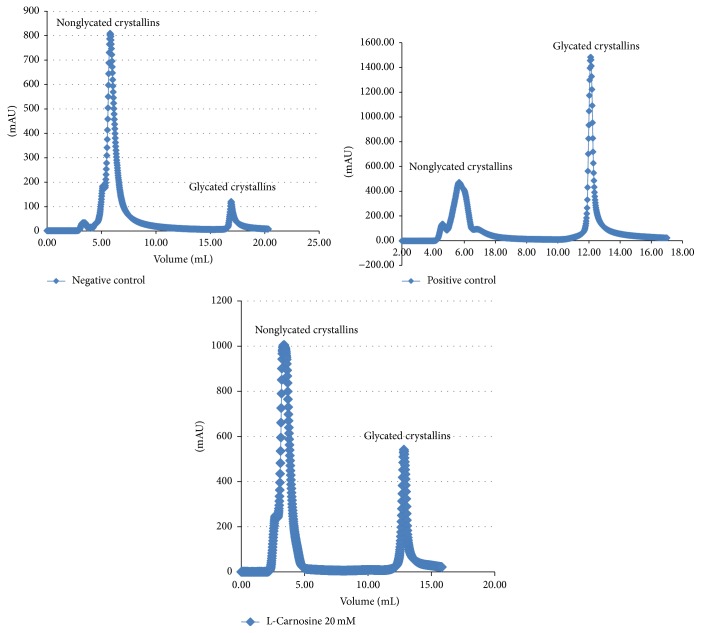
Boronate affinity chromatograms of nonglycated and glycated lens crystallins.

Approximately 15% of WS *α*-crystallins became insoluble or were degraded into lower molecular weight fractions as a result of lens incubation in the high galactose medium. By comparison, only 10% and 5% of WS *α*-crystallins were lost from lenses incubated in high galactose media with 10 and 20 mM L-carnosine, respectively (Figure 7). These results suggest that glycation may be linked with a loss of chaperone activity of *α*-crystallin. This concurs with previous work that found *α*-crystallins from goats lenses incubated with fructose and glucose-6-phosphate had a significant decrease in chaperone activity due to nonenzymatic glycosylation of *α*-crystallins [43]. It is worth noting that the galactose treatment did not show any notable changes in profiles of the WS fractions from size exclusion chromatography (Figure S2). This is because the study investigated early stage glycation which does not produce sufficient cross-linking that would manifest as large aggregates that would alter the size exclusion chromatographic profiles. The more sensitive test for these early stage changes is evident in affinity chromatography as shown in Figure 8.

The percentage of lens protein glycation recorded for the positive control was at least 30%, whereas treatment with L-carnosine showed a significant inhibition in the percentage of glycated proteins. Figure 8 shows separation of glycated crystallins from nonglycated crystallins using a boronate affinity chromatographic column for three different lenses incubated in media alone (negative control), positive control (media supplemented with 30 mM galactose), and the treated group with L-carnosine (20 mM). The negative control lenses showed the smallest fraction of glycated crystallins which represent physiologically normal glycated crystallins, whereas a sharp and intense glycated peak was recorded for positive control lenses at the expense of the nonglycated crystallin fraction. L-Carnosine treated lenses showed marked suppression of the glycated peak compared with the positive control. The glycated peaks recorded for L-carnosine-treated groups represent a combination of the physiological glycation fraction and a residual fraction of glycated proteins arising from the presence of galactose. These findings suggest a chaperone function of L-carnosine in protecting lens crystallins by inhibiting the formation of AGEs and accord with previous findings that show L-carnosine is a potent antiglycating agent [26].

### 3.7. Cytotoxicity Evaluation (MTT Assay) of L-Carnosine Using Human Lens Epithelial Cells

Table 1 shows estimated cell viability after a 3-hour exposure to different treatments. The positive controls (BKC and H_2_O_2_) have been shown to be cytotoxic over the duration tested in this study [44–46]. Three different concentrations of L-carnosine were examined for any cytotoxicity; these concentrations were low concentration 0.1% w/v (4.4 mM), 0.5% w/v (22 mM) as a middle drug concentration, and the highest concentration of 1% w/v (44.2 mM). The latter concentration is used commercially in the form of prodrug eye drops of N-acetyl L-carnosine. This concentration showed a significant cytotoxic (*P* < 0.05) effect; however, such a concentration is unlikely to reach the lens due to excessive drug loss and tear dynamics associated with topical ocular drug administration on the surface of the eye [47]. Whilst there were slight decreases in cell viability after exposure to lower concentrations 0.5% and 0.1% w/v, these were not statistically significant compared to the negative control (*P* > 0.05). It is worth nothing that BKC (which is cytotoxic when used over comparable duration) is used routinely as a preservative in ophthalmic eye drops at a concentration of 0.01% w/v; the duration of exposure of the eye to BKC when applied in eye drops is considerably shorter. These findings indicate that L-carnosine in the concentrations and for the durations tested does not demonstrate cytotoxicity in human lens epithelial cells.

## 4. Conclusion

L-Carnosine showed a potent antiglycation effect, in vitro, in cultured porcine lenses and human lens epithelial cells models. However, there is no evidence to support either direct or indirect antioxidant activities. Furthermore, using L-carnosine when there is no evidence of diabetes or of conditions that may induce such effects may cause harm to the redox glutathione system of the lens. These findings warrant investigations on the potential of L-carnosine as a therapeutic agent for delaying the development of diabetic cataract by mopping up excess glucose in the aqueous humour without deleterious effects on lens crystallins.

## Supplementary Material

S1: Fluorescence intensities for the different sample compounds showing (a) the details of each compound labelled from A – H and (b) the fluorescence intensities for compounds A-D and F-E and H-G. The subtraction of fluorescence intensity of the crystallins+glucose+L-carnosine combination (F and H) from glucose + L-carnosine (E and G respectively) is shown to indicate that the action of L-carnosine is as a carbonyl scavenging agent which competes for binding to glucose and thereby reduces the glycation of crystallin proteins.
S2: Size exclusion (SE) chromatograms showing: (A) porcine lens crystallins alone and (B) porcine lens crystallins incubated with galactose (30 mM). There is very little difference between the two profiles indicating that early stage glycation does not produce sufficient cross-linking in the water soluble fraction to induce an increase in the proportion of high molecular weight aggregates.

## Figures and Tables

**Table 1:** Immortalised human lens epithelial cell viability (%) after 3-hour exposure to the test substances employing MTT assay. Results are presented as mean values ± SD, *n* = 3.

Test substance	Lens cell viability (%)	Statistical significance to the medium
Media (negative control)	100	—
BKC (positive control)	17 ± 3.2	*P* < 0.05
H_2_O_2_ (positive control)	16 ± 3.6	*P* < 0.05
L-Carnosine (1%)	76 ± 13	*P* < 0.05
L-Carnosine (0.5%)	81.5 ± 9.2	*P* > 0.05
L-Carnosine (0.1%)	100 ± 10	*P* > 0.05
