# Supplementary figures and images for: On the Anticataractogenic Effects of L-Carnosine: Is It Best Described as an Antioxidant, Metal-Chelating Agent or Glycation Inhibitor?
Source: Oxid Med Cell Longev. 2016 Oct 16;2016:3240261. doi: 10.1155/2016/3240261 (PMC5086400; doi:10.1155/2016/3240261)

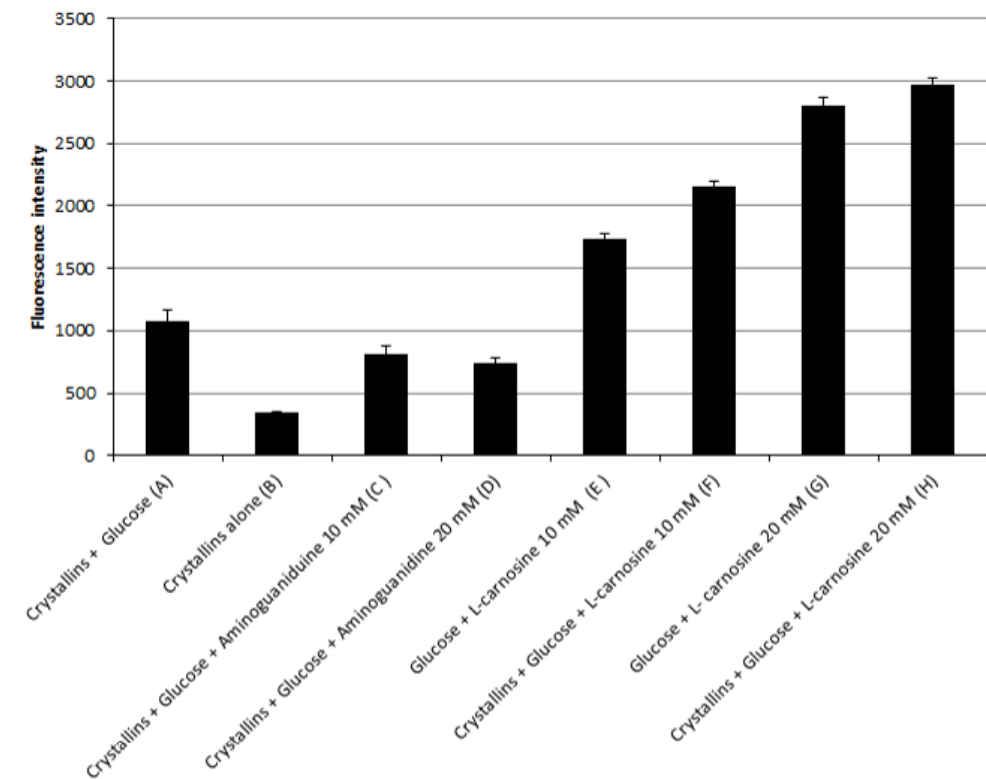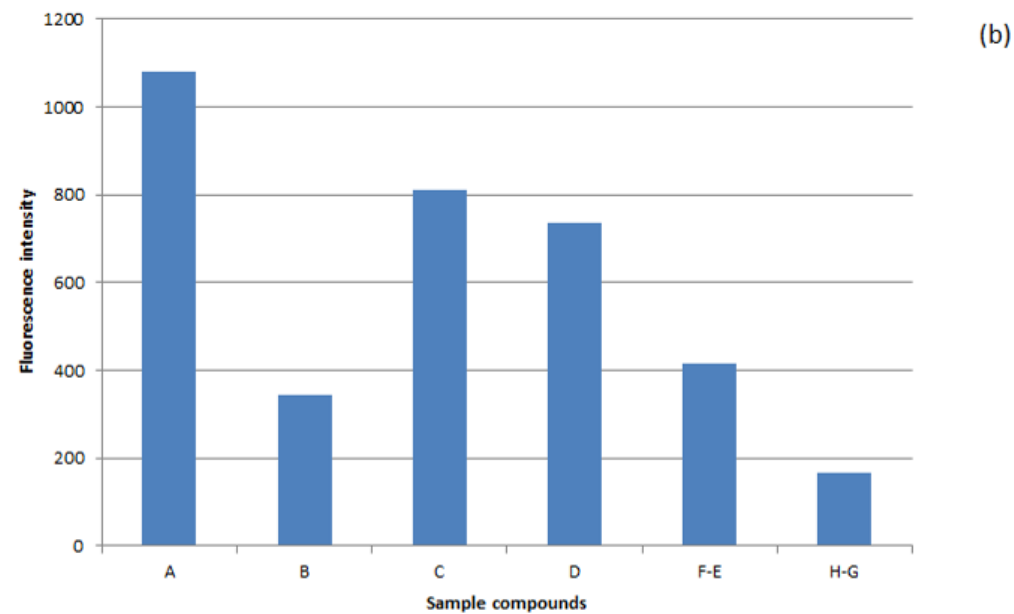

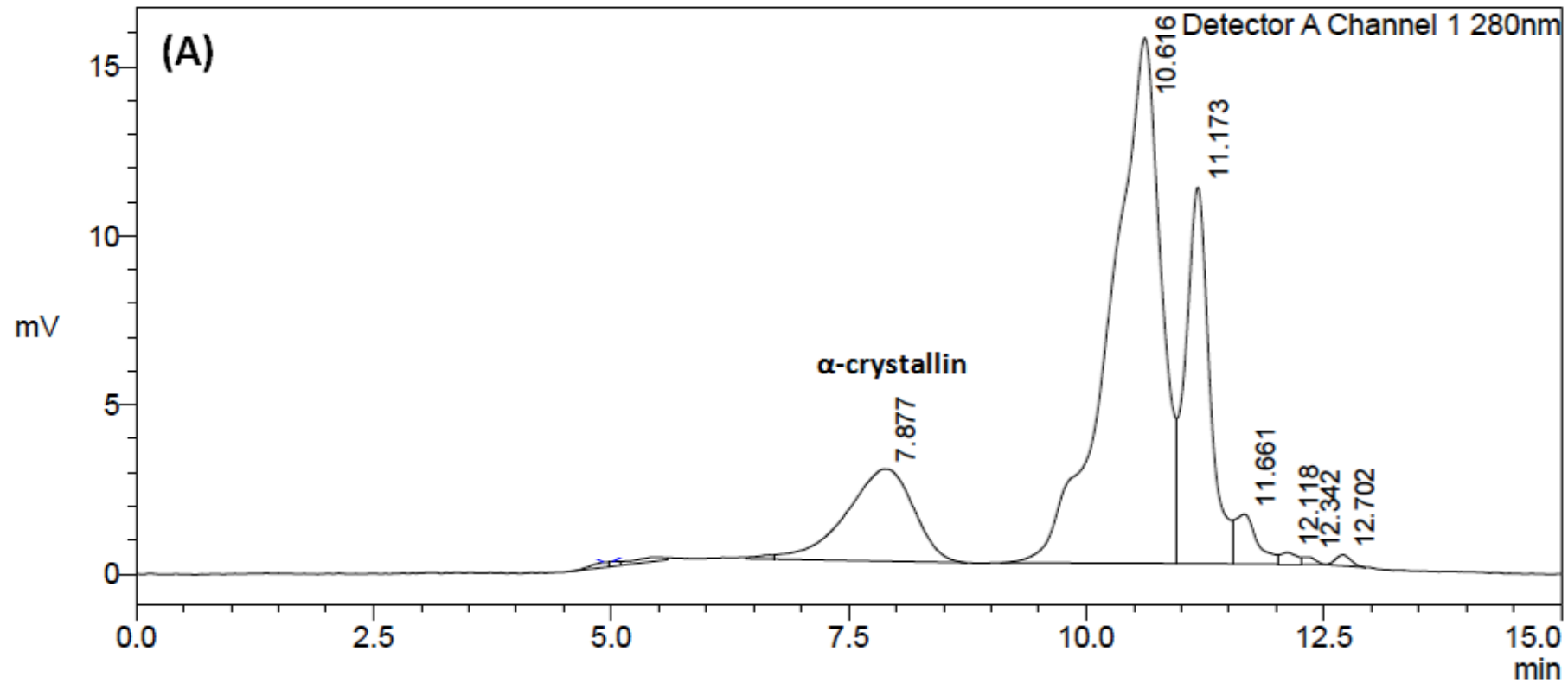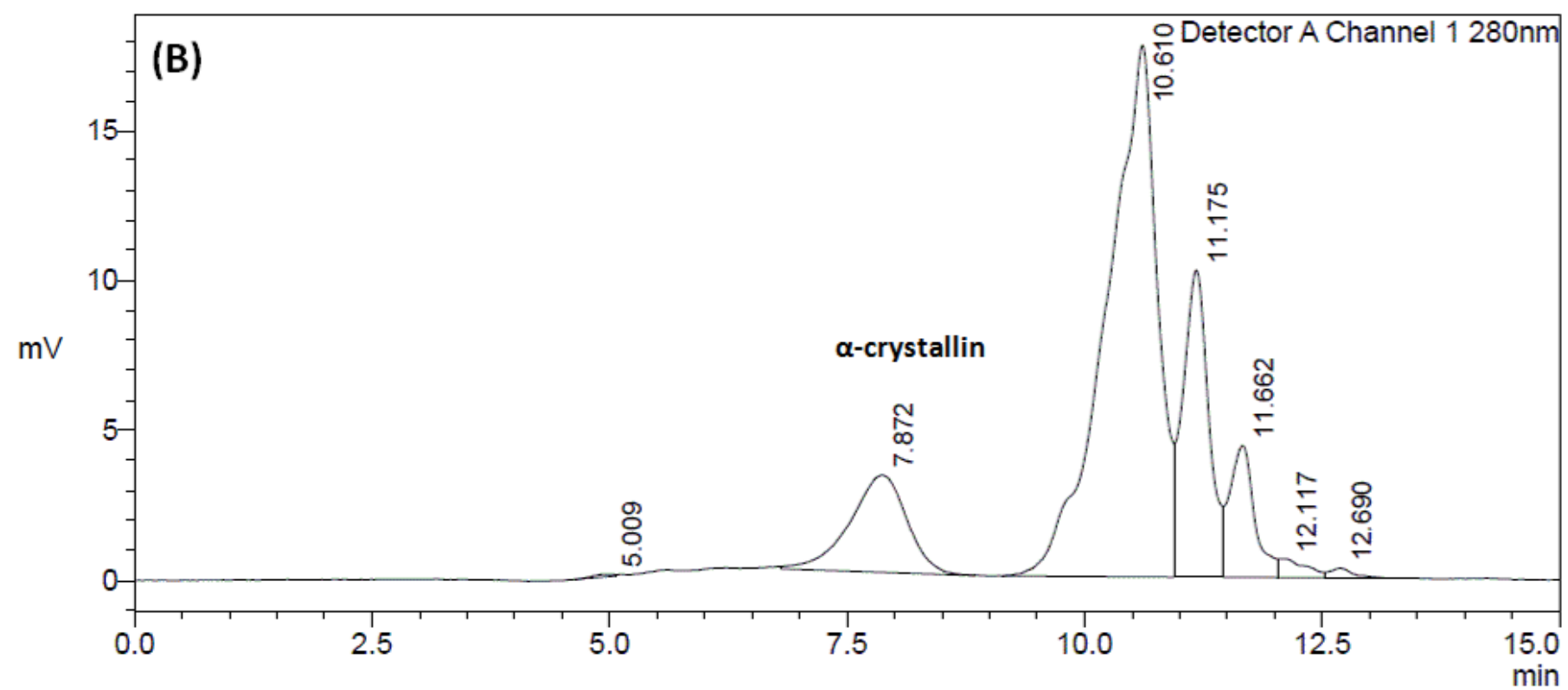

Supplement: Supplementary file 1 — S1: Fluorescence intensities for the different sample compounds showing (a) the details of each compound labelled from A – H and (b) the fluorescence intensities for compounds A-D and F-E and H-G. The subtraction of fluorescence intensity of the crystallins+glucose+L-carnosine combination (F and H) from glucose + L-carnosine (E and G respectively) is shown to indicate that the action of L-carnosine is as a carbonyl scavenging agent which competes for binding to glucose and thereby reduces the glycation of crystallin proteins. S2: Size exclusion (SE) chromatograms showing: (A) porcine lens crystallins alone and (B) porcine lens crystallins incubated with galactose (30 mM). There is very little difference between the two profiles indicating that early stage glycation does not produce sufficient cross-linking in the water soluble fraction to induce an increase in the proportion of high molecular weight aggregates. [file 3240261.f1.pdf]
